# Interactions between gold nanoparticles with different morphologies and human serum albumin

**DOI:** 10.3389/fchem.2023.1273388

**Published:** 2023-10-19

**Authors:** Jiahui Dai, Chao Chen, Man Yin, Huixing Li, Wenbo Li, Zhaowei Zhang, Qian Wang, Zhongyu Du, Xiangyu Xu, Yunfei Wang

**Affiliations:** ^1^ Clinical Medical College, Jining Medical University, Jining, Shandong, China; ^2^ Program for Scientific Research Innovation Team in Precision Medicine of Gynecologic Oncology, Affiliated Hospital of Jining Medical University, Jining, Shandong, China; ^3^ Laboratory of New Antitumor Drug Molecular Design and Synthesis, College of Basic Medical, Jining Medical University, Jining, Shandong, China; ^4^ School of Pharmaceutical Sciences, Wenzhou Medical University, Wenzhou, Zhejiang, China

**Keywords:** gold nanospheres, gold nanorods, gold nanoflowers, human serum albumin, fluorescence quenching, thermodynamic parameters

## Abstract

**Introduction:** Three different shapes of gold nanoparticles were synthesized in this experiment. At the same time, studies compared their effects with human serum albumin (HSA).

**Methods:** Gold nanoparticles (AuNPs) with three different morphologies, such as, nanospheres (AuNSs), nanorods (AuNRs), and nanoflowers (AuNFs) were synthesized via a seeding method and their characteristic absorption peaks were detected using ultraviolet-visible (UV-vis) absorption spectroscopy, Telectron microscopy (TEM), Dynamic Light Scattering (DLS) and Zeta potential measurements, circular dichroism (CD), and Fourier transform infrared spectroscopy (FTIR) to study the interactions between them and HSA. By comparing the thermodynamic parameters and quenching mechanism of the three materials, similarities and differences were determined in their interactions with HSA.

**Results:** The results showed that with an increase in the concentration of the AuNPs with the three different morphologies, the UV-vis absorption peak intensity of the mixed solution increased, but its fluorescence intensity was quenched. This indicates that the three types of AuNPs interact with HSA, and that the interactions between them represent a static quenching process, which is consistent with the conclusions derived from three-dimensional fluorescence experiments. Through variable-temperature fluorescence experiments, the binding constants, number of binding sites, and thermodynamic parameters of the interactions between the three types of AuNPs and HSA were determined. The Gibbs free energy changes were <0, indicating that the reactions of the three types of AuNPs with HSA are spontaneous, resulting in associated matter. Binding constant measurements indicated that the strongest binding took place between the AuNFs and HSA. In addition, the results of fluorescence, CD spectroscopy, and FTIR showed that three different shapes of AuNPs can induce conformational changes in HSA and reduce the α-helix content. Among them, AuNFs have the smallest ability to induce conformational changes.

**Discussion:** According to studies, AuNFs interact more favorably with HSA. This can be used as a reference for the administration of drugs containing AuNPs.

## 1 Introduction

Nanoscale metal materials were first successfully developed in the mid-1980s. With the development of science and technology, a variety of nanomaterials, such as nano-biomedical materials and nano-magnetic materials, have attracted a great amount of research attention (Baetke et al., 2015). Nanotechnology is a research field that holds great potential, and progress made in this area has enabled various types of nanoparticles (NPs) to be used in clinical diagnosis and treatment. Most of the types of NPs developed are currently in clinical use for therapeutic purposes (Baetke et al., 2015; Alkilany et al., 2019). Nanomaterials that have been studied the most include gold (Ahmad et al., 2013), silver (Lee and Jun, 2019), and iron oxide nanomaterials (Shi et al., 2015). Gold was one of the first metals discovered, therefore meaning that it has already been researched and used in applications for more than a thousand years. Recently, the research on gold nanomaterials has attracted much attention (Daraee et al., 2016). AuNPs are widely used in many fields, such as medicine, biology, and chemistry, due to their unique physical and chemical properties (Fan et al., 2014; Quader and Kataoka, 2017), which manifest in the following ways (Panahi et al., 2017; Zheng et al., 2017; Bai et al., 2020): 1) AuNPs show supramolecular and molecular recognition characteristics, and 2) AuNPs have unique electrical, optical, magnetic, catalytic, and biological affinity effects. Many studies have shown that AuNPs with different morphologies exhibit different properties (Jackson et al., 2011; Haine and Niidome, 2017; Mao et al., 2020), with the most representative being AuNSs, AuNRs, and AuNFs. Due to the excellent optical and catalytic properties of these materials, they have broad application prospects in the fields of chemistry, biology, and medicine. Photothermal therapy, based on the interactions between AuNPs and proteins, can be used in therapy on the human body. The research demonstrated that plasma photothermal therapy-based biomimetic keratin-embedded AuNPs are extremely effective photosensitive nanotherapeutic agents, which are the against glioblastoma multiforme (Guglielmelli et al., 2020). Photothermal therapy (Park et al., 2019) is a method that involves the conversion of light energy to heat energy to inhibit the rapid growth of tumors. AuNPs are a photothermal agent that exhibits good biocompatibility. When it comes to AuNPs, AuNRs are a typical representation employed in tumor photothermal therapy since they can be activated by near-infrared light. Based on the phenomenon of localized surface plasmon resonance, it produces a thermal response to near-infrared lasers that causes the temperature to rise quickly to 50°C to 70°C, exceeding the threshold that kills tumor cells and tumor capsule blood vessels while barely affecting the function of normal cells. Moreover, irradiation period, laser intensity, and AuNPs concentration can all be used to effectively adjust the temperature of AuNPs exposed to a near-infrared laser (Zhang et al., 2018; Park et al., 2019; Nejabat et al., 2023). Infrared light is used to excite nanoparticles that have resonance in that area of the electromagnetic spectrum. NIR light is typically employed because it falls within the biological window and enables you to penetrate the body more deeply. It is also possible to use wavelengths with reduced tissue penetration for superficial or skin cancers. Its use *in vivo* photothermal therapy of superficial cancers, such as malignant melanoma (Zhang et al., 2018; Campu et al., 2020) and breast cancer (Au et al., 2008; Mokoena et al., 2019), is made possible by this feature, and it has produced impressive results. Protein-coated AuNPs are effective photosensitive nanotherapeutic agents for plasma photothermal therapy and have good biocompatibility, efficient cellular uptake, and local photothermal capabilities, according to more pertinent publications published in recent years (Guglielmelli et al., 2020).

Protein is a ubiquitous component of all of the cells and tissues in the human body. Thus, all of the important parts of the human body require the participation of protein. Proteins have complex three-dimensional (3D) structures, which is important for their biological and immune activity (Mokaberi et al., 2021). Human serum albumin (HSA) is the most abundant protein in human plasma, where it has the functions of transporting fatty acids, amino acids, drugs and other substances, and maintaining plasma osmotic pressure. Due to its wide presence in blood and easy purification, HSA became one of the earliest and most widely studied proteins (Lee et al., 2017). In clinical treatment, it is used to treat diseases such as burns and shock (Mokaberi et al., 2021). It is known that in order for medications to effectively cure diseases *in vivo*, they must first travel through the circulatory system to reach the intended tissues. HSA can be employed as a drug transport carrier since it is the most prevalent and significant soluble protein component in human blood. It also has the ability to bind to a variety of endogenous and exogenous ligands in the blood (Naveenraj et al., 2010).

For a long time, scientists have focused their attention on the interaction between AuNPs with proteins (Li et al., 2022). AuNPs provide a good surface for proteins (Naveenraj et al., 2010; Abdelhamid and Wu, 2016). Proteins and other biomolecules are thought to swiftly form a “protein corona” on the surfaces of nanoparticles when they are exposed to biological settings (Cedervall et al., 2007; Guglielmelli et al., 2023). Protein adsorption intensity is influenced by the nanosurface’s characteristics as well as the kind of serum protein. Compared to other plasma whites, HSA and fibrinogen have the highest rates of binding and dissociation (Cedervall et al., 2007). Several of the characteristics of nanoparticles, such as size, dispersion, stability, surface charge, chemical properties, etc., will also alter after they are coupled with serum proteins. Furthermore, this might provide such composites additional biological properties, which is more significant (Gao et al., 2005; Naveenraj et al., 2010; Awotunde et al., 2020; Simon et al., 2021; Wen et al., 2022; Yang et al., 2022). AuNPs can be employed as medication carriers because of their strong affinity for HSA, according to research by Naveenraj et al. (2010). The presence of HSA considerably increased both the biocompatibility of metal nanoparticles and the light-induced effect of AuNPs, according to the study of Guglielmelli et al. Further research has proven that serum proteins and cationic AuNPs work together to greatly lessen the toxicity of the latter (Wen et al., 2022).

Due to their tiny size (which enables capillary transport), great chemical stability, strong biocompatibility, ease of multifunctional surface modification, and tunable optical features, gold nanoparticles have a wide range of *in vivo* application potential (Ravindran et al., 2010; Dheyab et al., 2022; Yang et al., 2022). It is crucial to compare AuNPs of various forms since theoretical and experimental investigations have demonstrated that the size and shape of AuNPs significantly affect the aforementioned qualities (Dheyab et al., 2022). In this study, AuNSs, AuNRs, and AuNFs were synthesized via chemical synthesis and characterized. Methods such as spectroscopy were used to study the interactions between the three different types of AuNPs and HSA. The corresponding thermodynamic parameters were obtained for comparison and analysis. Overall, the results provide a theoretical basis for the application of AuNPs with different morphologies in the field of medicine.

## 2 Experimental section

### 2.1 Materials

Electronic balance (EL 204, METTLER TOLEDO Instruments Co., Ltd., Shanghai), UV-vis spectrophotometer (Shimadzu UV-2501PC, Japan), fluorescence photometer (Hitachi F-4600, with a constant temperature sample holder, model 250 -0346, Japan), German Eppendorf adjustable pipette, YouPu series ultrapure water device (UPD-II-10T, Sichuan), Circular dichroism spectrometer (JASCO-810 spectropolarimeter, Japan), Fourier Transform Infrared Spectrometer (PE/Frontier, the United States), Nanoparticle size and Zeta Potentiometer (Litesizer 500, Austria) and Transmission electron microscopy (Talos L120C TEM, Czech Republic).

Trisodium citrate dihydrate (Na_3_C_6_H_5_O_7_) was purchased from Tianjin Bodi Chemical Co., Ltd.; chloroauric acid (HAuCl_4_) was purchased from Sinopharm Chemical Reagent Co., Ltd.; ascorbic acid (AA) was purchased from Beijing Bailingwei Technology Co., Ltd.; boron sodium hydride (NaBH_4_, purity 98%) and tri-methylolaminomethane (Tris) were purchased from Alfa; cetyltrimethylammonium bromide (CTAB, C_19_H_42_NBr, purity 99%) was purchased from Beijing Xinke Zhongjing Biotechnology Technology Co., Ltd.; and silver nitrate (AgNO_3_) and HSA (fatty acid-free, purity 96%–99%, *M*_w_ = 66,500 g·mol^−1^) were both purchased from Beijing Xinke Zhongjing Biotechnology Co., Ltd. The HSA solution was stored in a refrigerator at 4°C prior to its use. All of the water used in the experiments was deionized three times before use.

### 2.2 Preparation of AuNPs

#### 2.2.1 Preparation and characterization of the AuNSs

To a beaker, chloroauric acid (20 mL, 2.5 × 10^−4^ mol/L) and trisodium citrate (2.5 × 10^−4^ mol/L) were added. Then, to this, 0.1 mol/L of ice-cold NaBH_4_ solution (0.6 mL) was added while stirring. Afterwards, the solution mixture immediately turned pink, indicating that gold seeds were formed. This solution was then used as a seed solution within 2–5 h. In this experiment, citrate was used as an end-capping agent, and the diameter of the gold seeds was 3.5 nm.

The preparation of the AuNPs involved a three-step seeding method. Three test tubes were labeled A, B, and C, each containing 9 mL growth solution, consisting of 2.5 × 10^−4^ mol/L HAuCl_4_ and 0.1 M CTAB. AA (0.05 mL of a 0.1 mol/L solution) was then added to each test tube. Then, gold seed solution (1.0 mL) was mixed with sample A, the color of which turned red within 2–3 min. After 4–5 h, 1.0 mL of solution from test tube A was taken out and added to test tube B, which was then mixed well. Solution B turned red within 4–5 min, after which 1.0 mL of solution was taken from test tube B and added to test tube C, which was then mixed well. Solution C turned red within 10 min, with the resulting solution being AuNSs.

Preliminary characterization of the synthesized AuNSs was carried out using a UV-vis spectrophotometer and TEM.

#### 2.2.2 Preparation and characterization of the AuNRs

AuNRs were prepared via a seed-induced growth method. In this method, a 1% HAuCl_4_ solution (0.103 mL) was added to a 0.1 mol/L solution of CTAB (10 mL), and a 0.01 mol/L ice-cold solution of NaBH_4_ (0.6 mL) under vigorous stirring. The solution mixture was then stirred for 2 min to obtain a AuNPs seed solution.

A 1% solution of AgNO_3_ (1.2 mL), a 1% solution of HAuCl_4_ (1.03 mL), and a 0.0788 mol/L solution of AA (0.35 mL) were added sequentially to a 0.1 mol/L solution of CTAB (50 mL) to prepare a growth solution. Then, seed crystals (0.06 mL) were added to the growth solution, which was shaken slowly to ensure its uniform mixing, and it was then left to stand in a water bath at 30°C for 6 h to prepare uniformly sized AuNRs.

Preliminary characterization of the synthesized AuNRs was carried out using an UV-vis spectrophotometer and TEM.

#### 2.2.3 Preparation and characterization of the AuNFs

Preparation of the AuNFs was carried out using an AA reduction method. To prepare spherical gold seeds, a 0.25 mmol/L solution of HAuCl_4_ solution (100 mL) was added to a round-bottom flask, which was heated to the point of boiling under stirring, to which a 5% (mass fraction) aqueous solution of sodium citrate (1 mL) was added. After 5 min the heating was stopped, as a deep red solution was produced containing AuNSs of around 25 nm in size. The above solution was centrifuged at 10,000 r/min for 15 min, the supernatant removed, and the precipitate was dispersed in ultrapure water of the same volume as that of the original solution. The solution was centrifuged again under the same conditions to remove the supernatant, and the precipitate was dispersed in ten times the volume of ultrapure water as the original solution and was stored for later use.

To prepare AuNPs, a 0.2 mol/L aqueous solution of AA (7.5 μL) was added to a stock solution of gold seeds (2 mL) while stirring at room temperature, and then, to this, a 8.62 mmol/L solution of HAuCl_4_ (58.0 μL) was added. Within 5 s, the color of the solution changed rapidly from pink to purple–red, and finally, blue–purple.

Preliminary characterization of the synthesized AuNFs was carried out using a UV-vis spectrophotometer and TEM.

### 2.3 Characterization techniques

#### 2.3.1 Spectrophotometric measurements

Using a UV-vis spectrophotometer, UV-vis absorption spectra of the solution mixtures in the range of 200–900 nm were measured at a scan rate of 1,200 nm/min.

#### 2.3.2 TEM characterization

Put AuNSs and AuNRs into a centrifuge, centrifuge at 10,000 r/min for 15 min, and then remove the supernatant. Add an equal amount of ultrapure water to the original solution into the centrifuge tube, centrifuge again under the above conditions, and remove the supernatant. Then, disperse the precipitate in an equal amount of ultrapure water as the original solution. Take appropriate amounts of three samples: AuNSs, AuNRs, and AuNFs, and drop them on the copper grid. After drying, the copper mesh carrying the sample was subjected to TEM observation.

#### 2.3.3 DLS and Zeta potential measurements

Take 1 mL of AuNSs, AuNRs, and AuNFs solutions, respectively. After diluting the sample with 1 mL of deionized water, use a particle size and Zeta potential meter to measure the particle size and Zeta potential. Use dynamic light scattering software to record the average particle size and surface of the nanoparticles. Potential. Again, take 1 mL of AuNSs, AuNRs, and AuNFs solutions, add 1 mL of HSA solution, and repeat the above detection.

#### 2.3.4 FTIR spectroscopy measurements

Take 1 mL of AuNSs, AuNRs, AuNFs, and HSA solutions, respectively; dilute the samples with 1 mL of deionized water; and place them into 3 mL centrifuge tubes. Again, take 1 mL of AuNSs, AuNRs, and AuNFs, mix them with 1 mL of HSA solution, and put them into 3 mL centrifuge tubes. Take an appropriate amount of dry potassium bromide powder, put it into a mold, press it into tablets on a tablet press, drop 2.5 μL of the sample into each sample, wait for dryness, and detect. The band range is 4,000 to 400 cm^1^.

#### 2.3.5 CD spectroscopy measurements

CD measurements were recorded using a JASCO-810 spectropolarimeter. A quartz cell with a 0.1 cm path length was used in a nitrogen atmosphere. The CD spectra were operated over the range of 200–260 nm at room temperature.

#### 2.3.6 Fluorescence detection measurements

For fluorescence detection measurements, the excitation wavelength of the fluorescence spectrophotometer, was set to 280 nm, the corresponding scan width was set to 300–500 nm, and the width of the emission and excitation slits were both set to 5 nm. The obtained fluorescence data was processed to create Stern–Volmer plots, and the thermodynamic parameters of each binding system were calculated. Time-resolved fluorescence measurements were carried out using a FLS920 combined fluorescence lifetime and steady state spectrometer (Edinburgh, United Kingdom). HSA was fixed at a concentration of 0.3 mM and was excited at 280 nm.

## 3 Results and discussion

### 3.1 UV-vis spectrum analysis of the AuNSs, AuNRs, and AuNFs

**FIGURE 1:**
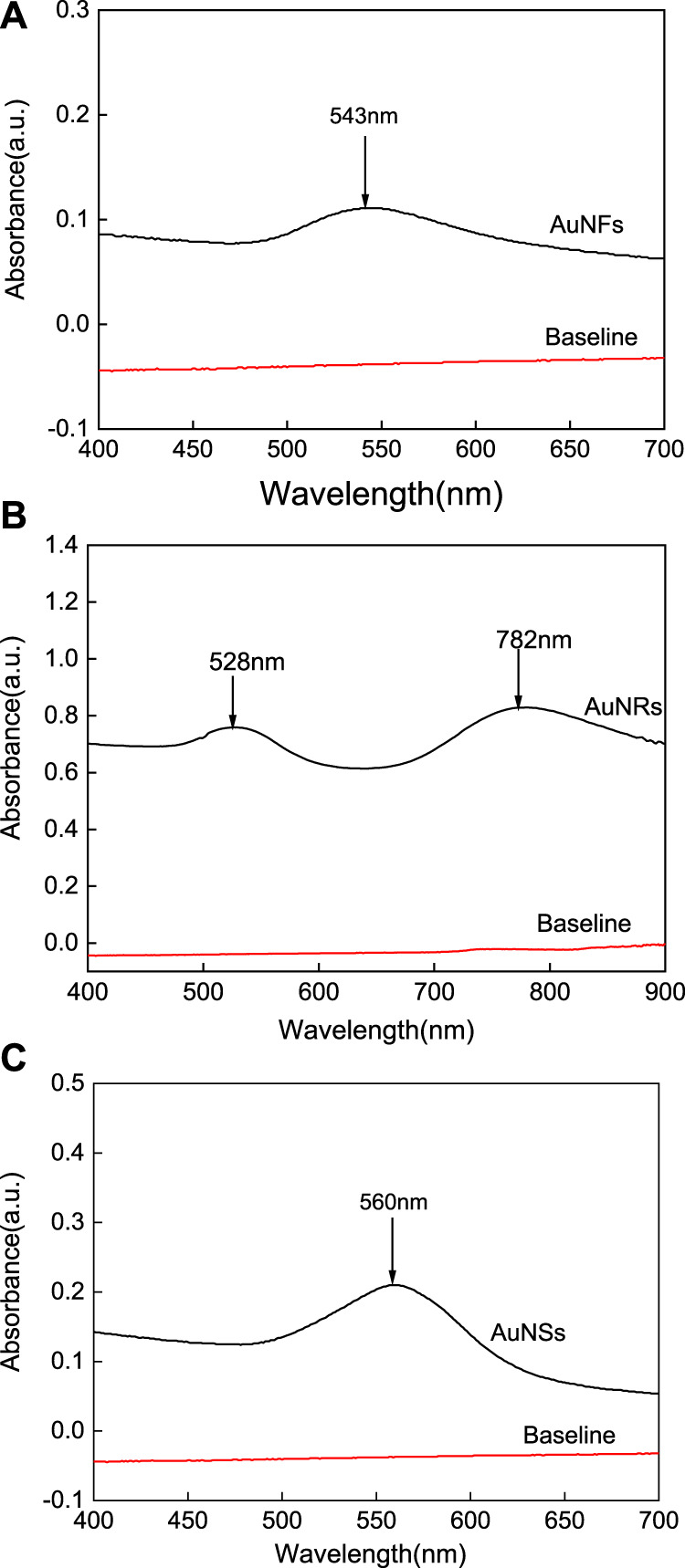
The UV-vis absorption spectra of three different shapes of the AuNPs: AuNFs **(A)**, AuNRs **(B)**, AuNSs **(C)**.

Metallic nanoparticles have plasmon resonance bands that can fall from UV to NIR based on composition, shape and size. Therefore, it is very meaningful to observe the optical phenomenon of gold nanostructures, as their absorption is related to their particle size, shape, and state of aggregation (Wang and Ni, 2014; Grand et al., 2019). Figure 1A shows the UV-vis spectrum of the AuNFs obtained in this study, from which it can be seen that the particles in solution exhibit an obvious UV-vis absorption peak at 543 nm, indicating that the solution contains flower-like particles. The ridges on the surface of the AuNFs are what are responsible for this distinctive absorption peak. Mie scattering theory states that AuNRs have two absorption peaks because of their extremely asymmetric shape: one in the 510–530 nm range, known as the transverse resonance peak, and one in the 600–900 nm visible to near-infrared light region, known as the longitudinal formant (Gao et al., 2003). Figure 1B shows the UV-vis spectrum of the AuNRs obtained in this study, from which it can be seen that the particles exhibit obvious UV-vis absorption peaks at 528 and 782 nm, indicating that the solution contains particles with a rod-shape morphology. The SPR of AuNSs reveals a single peak in the visible light region, in accordance with the Mie scattering hypothesis, because of its centrally symmetric shape. Figure 1C shows the UV-vis spectrum of the AuNSs obtained in this study, from which it can be seen that the particles in solution exhibit an obvious UV-vis absorption peak at 560 nm, indicating that they are spherical. In summary, these experiments were carried out to synthesize three types of AuNPs: AuNFs, AuNRs, and AuNSs.

### 3.2 TEM detection of nanomorphology

**FIGURE 2:**
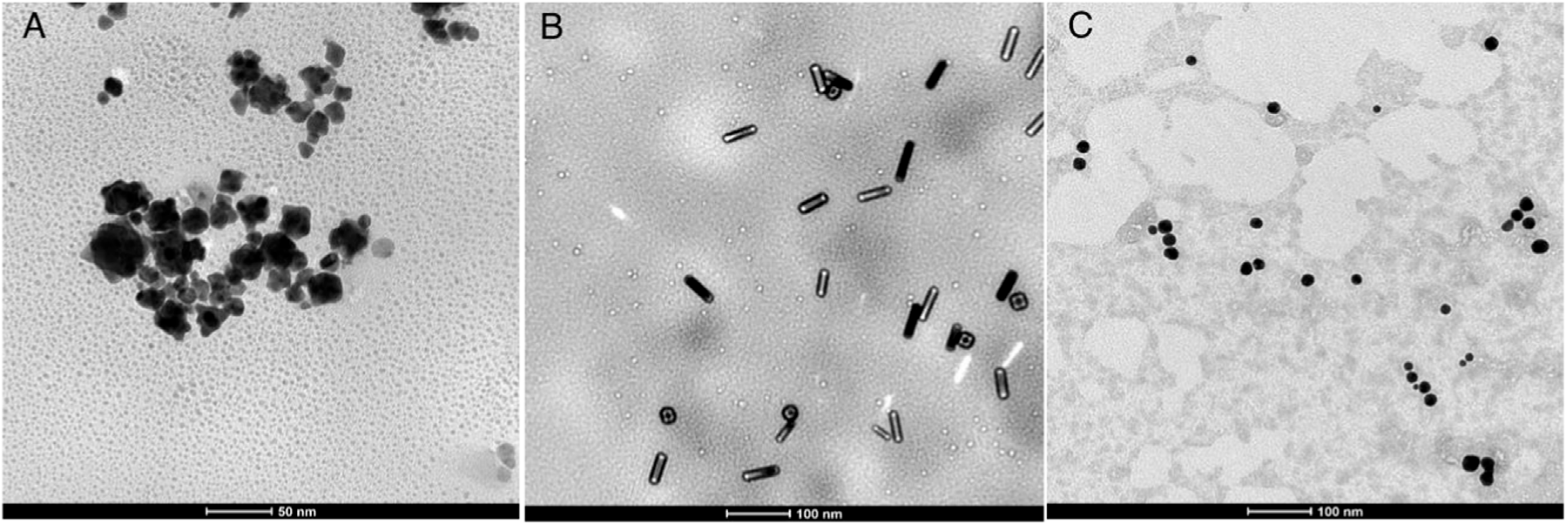
Typical TEM images of three different shapes of AuNPs: **(A)** AuNFs, **(B)** AuNRs, and **(C)** AuNSs.

**FIGURE 3:**
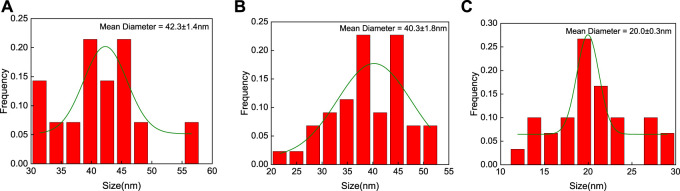
According to TEM analysis of the particle sizes of three different shapes of AuNPs, AuNFs **(A)**, AuNRs **(B)**, and AuNSs **(C)**, the average diameters are approximately 42.3 ± 1.4, 40.3 ± 1.8, and 20.0 ± 0.3 nm, respectively.

TEM can provide accurate responses to the morphology and size of synthesized nanomaterials. As shown in Figure 2, the three different shapes of AuNPs synthesized above were observed through TEM, showing flower-like, rod-like, and spherical shapes, and all had good dispersion. After measurement and analysis, the average diameters of AuNFs, AuNRs, and AuNSs are approximately 42.3 ± 1.4 nm (Figure 3A), 40.3 ± 1.8 nm (Figure 3B), and 20.0 ± 0.3 nm (Figure 3C), respectively.

### 3.3 DLS and Zeta potential measurements of the interactions between the AuNFs, AuNSs, AuNRs, and HSA

**FIGURE 4:**
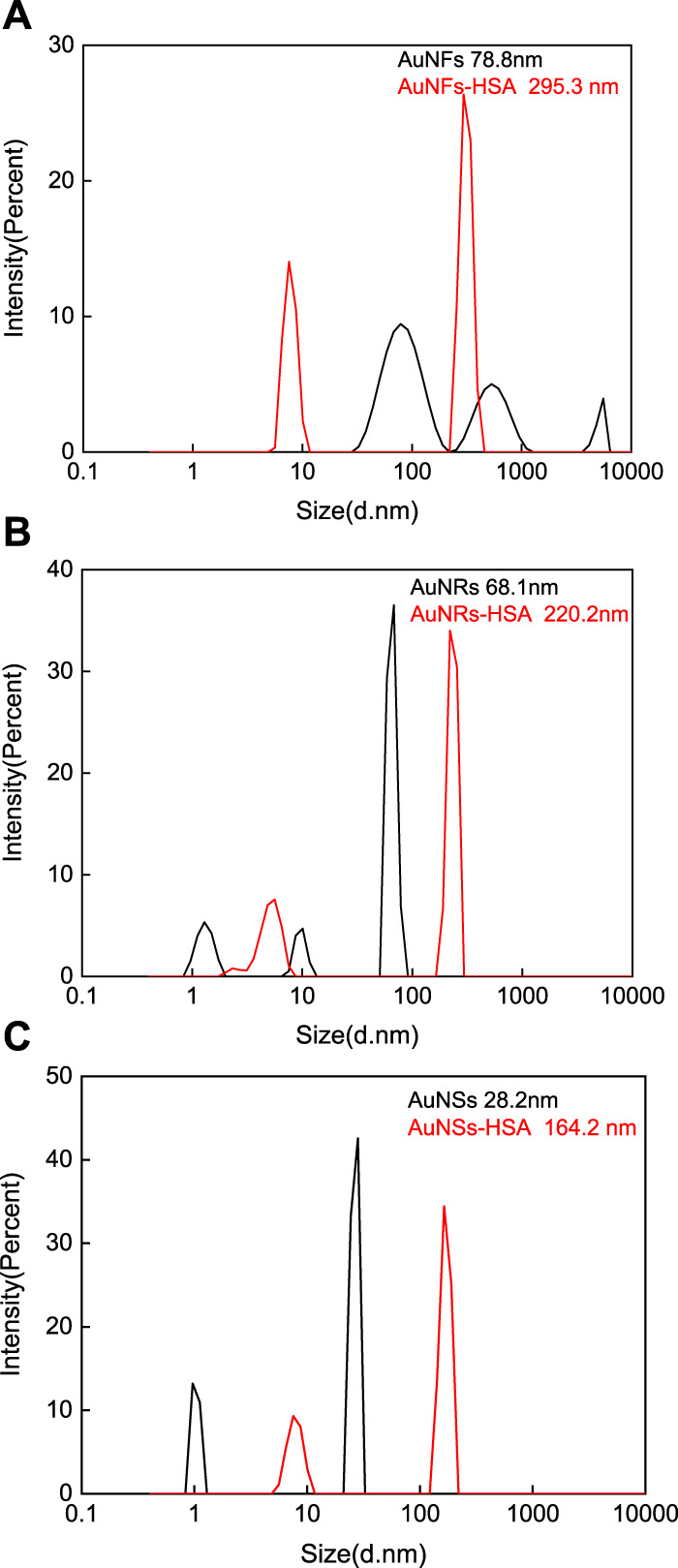
DLS measured the particle size changes of three different shapes of AuNPs before and after the interaction with HSA: **(A)** AuNFs, **(B)** AuNRs, and **(C)** AuNSs. (HSA) = 6.0 × 10^−6^ mol·L^−1^.

DLS and Zeta potential measurements were conducted before and after the interaction between three different shapes of AuNPs and HSA. This further verified the particle sizes of three different shapes of AuNPs and their particle size changes before and after the interaction with HSA. As shown in Figure 4, the average diameters of pure AuNFs, AuNRs, and AuNSs are approximately 78.8 nm (Figure 4A), 68.1 nm (Figure 4B), and 28.2 nm (Figure 4C), respectively. This is slightly larger than the results measured by TEM. The reason is that the particle size measured by DLS is the hydrated particle size, which is consistent with reality. After interacting with HSA, the average diameters of AuNFs-HSA, AuNRs-HSA, and AuNSs-HSA were approximately 295.3 nm (Figure 4A), 220.2 nm (Figure 4B), and 164.2 nm (Figure 4C), respectively. The DLS results clearly show that HSA can be adsorbed on the surface of AuNPs (Selva Sharma and Ilanchelian, 2015).

Through Zeta potential measurements, the potential of three different shapes of AuNPs changed after interacting with HSA. Simple AuNFs, AuNRs, and AuNSs all have positive charges on their surfaces, with average potentials of approximately 11.05, 64.03, and 60.60 mV, respectively. After interacting with HSA, the surfaces of AuNRs-HSA and AuNSs-HSA still carry positive charges, with average potentials of approximately 26.72 and 26.27 mV, respectively. However, the surface of AuNFs-HSA is negatively charged, with an average potential of about −9.03 mV. It was observed from the results that after the interaction with HSA, the surface charges of the three AuNPs showed a decrease in varying degrees. The above phenomenon is attributed to the negative charge on the surface of bound HSA, so its surface charge is reduced after binding to positively charged AuNPs, which is consistent with previous studies (Selva Sharma and Ilanchelian, 2015; Xie et al., 2017).

### 3.4 UV-vis spectrum analysis of the interactions between the AuNFs, AuNSs, AuNRs, and HSA

**FIGURE 5:**
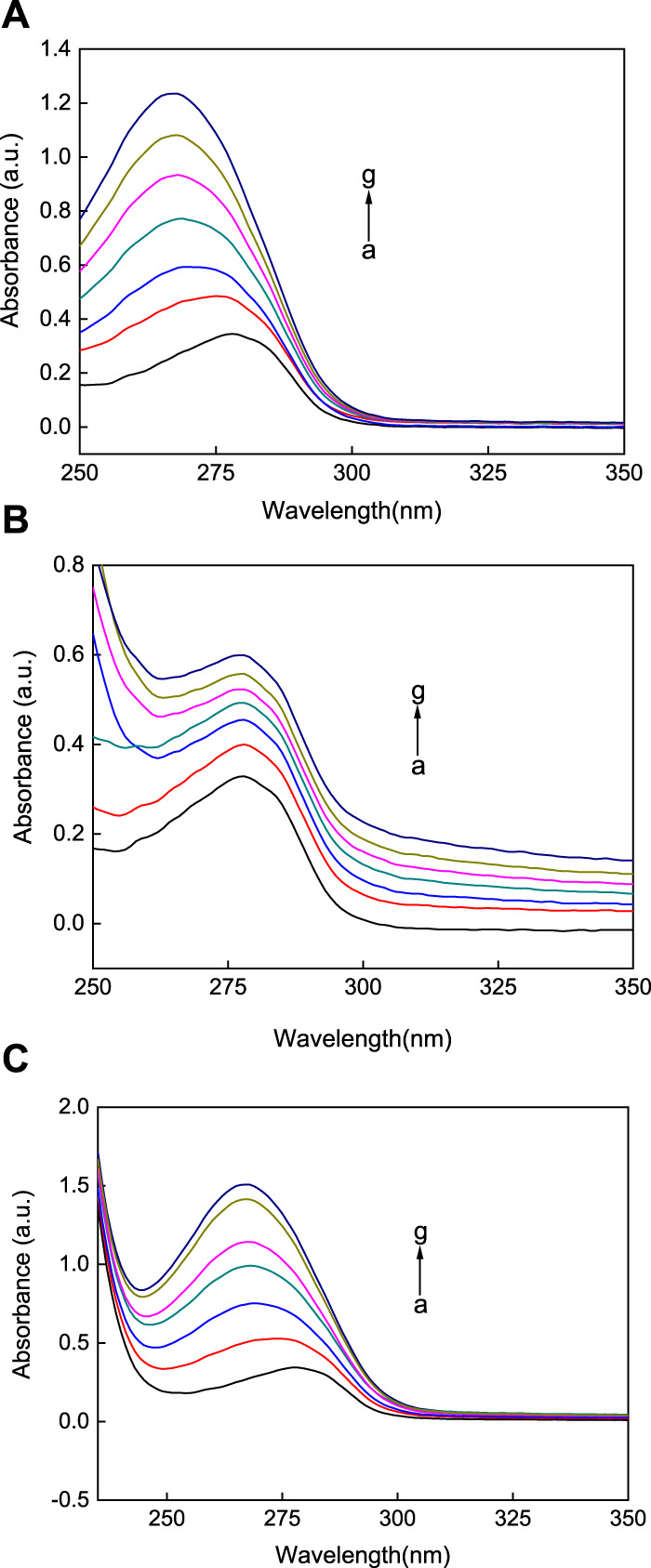
The UV-vis absorption spectra of three different shapes of the AuNPs **(A)** AuNFs, **(B)** AuNRs, **(C)** AuNSs binding with HSA. The concentration of HSA was fixed at 3.00 μM; the concentration of the AuNPs from (a) to (g) is 0.00, 8.33, 16.70, 25.00, 33.30, 41.70, and 45.80 μM.

UV-vis absorption spectroscopy is an important method that is used to study the interactions between the AuNPs and HSA (Li et al., 2020). In this work, UV-vis absorption spectroscopy was used to study the interactions between the AuNPs with the three different morphologies and HSA. As shown in Figure 5, the absorption peak of the blank HSA solution is around 280 nm. Upon the addition of the AuNPs solutions of different concentrations, the three different types of AuNPs led to a regular increase in the UV-vis absorption intensity of HSA. That is, with an increase in the concentration of the AuNPs with the three different morphologies, the sorbance of the HSA solution was enhanced. This technique offers trustworthy proof of changes in refractive index around the protein brought on by interactions with AuNPs. In addition, this experiment noted that observable plasma shifts happened as AuNFs and AuNSs concentrations rose. Figures 5A, C illustrates how the greatest value is blue-shifted (towards lower wavelengths). Nevertheless, with AuNRs, there was no discernible plasmon shift, as seen in Figure 5B.

### 3.5 FTIR spectral analysis of the interaction between AuNFs, AuNSs, AuNRs, and HSA

FTIR spectroscopy can effectively utilize protein structure-related resonance transitions to obtain information about protein conformational changes induced by AuNPs. FTIR is a powerful technique for determining the secondary structure of proteins without limiting their molecular weight. In FTIR studies of proteins, the focus is on assigning the different components of the secondary structure in amide I. The assignments recommended in most protein studies are as follows: 1,649–1,660 cm^−1^ (α-helix), 1,618–1,642 cm^−1^ (β-sheet), 1,666–1,688 cm^−1^ (turn), 1,618–1,623 cm^−1^ (intramolecular aggregate, A1), and 1,683–1,689 cm^−1^ band (intramolecular aggregate, A2), respectively (Shao et al., 2011; Zhang et al., 2012).

**FIGURE 6:**
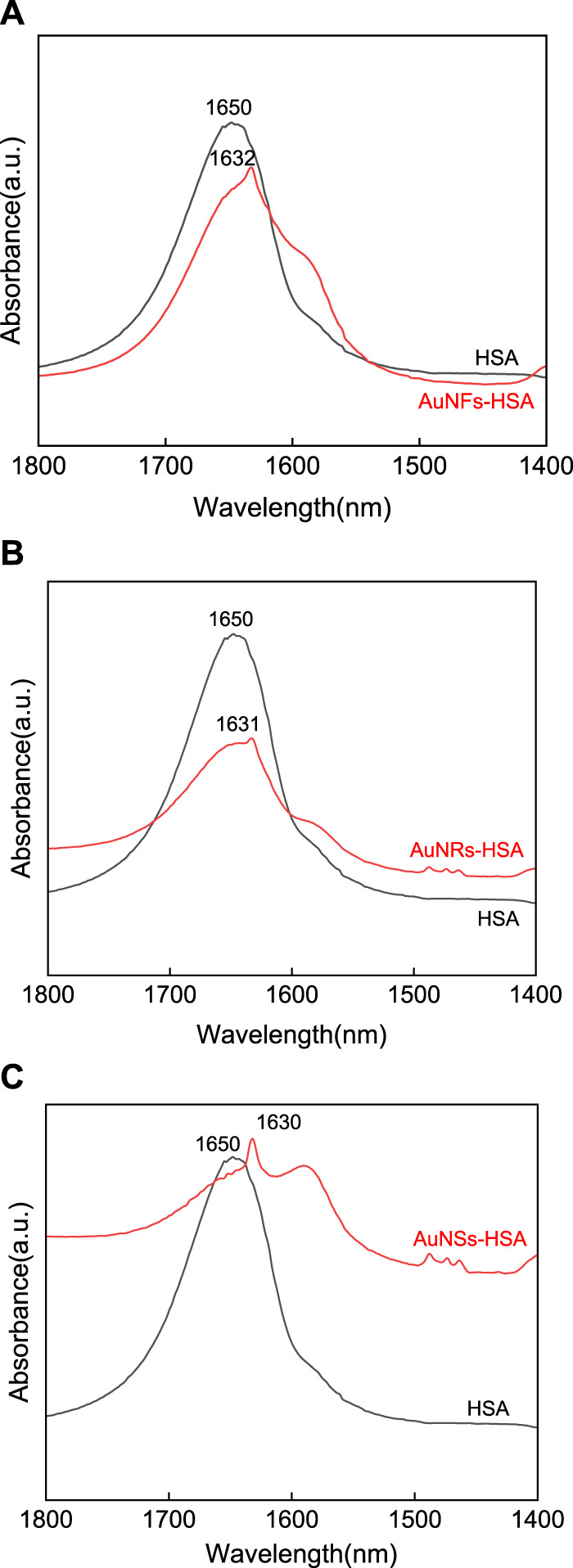
FTIR Spectral analysis of the interaction between **(A)** AuNFs, **(B)** AuNRs, **(C)** AuNSs, and HSA, respectively. (HSA) = 6.0 × 10^−6^ mol·L^−1^.

The original infrared spectrum of HSA (see Figure 6), in which a strong band centered at 1,651 cm^−1^ can be observed in the amide I region, shows that the HSA conformation is rich in α-helix. Compared with HSA, the amide I bands in AuNFs-HSA (Figure 6A), AuNRs-HSA (Figure 6B), and AuNSs-HSA (Figure 6C) bioconjugated systems showed obvious differences in shape and peak position (1,649–1,660 cm^−1^), which indicates that the secondary structure of HSA has changed in the bioconjugation system. Meanwhile, it can be found that the three different shapes of AuNPs induce slightly different degrees of changes in the HSA secondary structure. After adding AuNFs, the peak position moved from 1,650 to 1,632 cm^−1^. After adding AuNRs, the peak position moved from 1,650 to 1,631 cm^−1^. After adding AuNSs-HSA, the peak position moved from 1,650 to 1,631 cm^−1^. The order from small to large is AuNFs, AuNRs, and AuNSs. The above results have also been confirmed for other nanometers. According to the provious studies, it can be known that the FTIR spectroscopy analysis was similar to our results when studying the interaction between silver nanometers of different shapes and BSA (Zhang et al., 2023).

### 3.6 CD spectroscopy analysis

**FIGURE 7:**
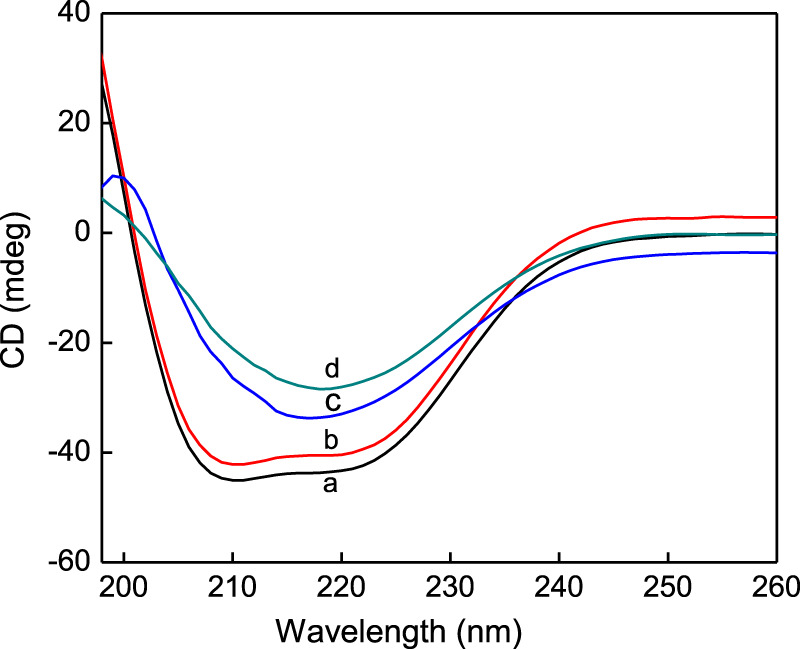
CD spectra of pure HSA (curve a), AuNFs-HSA (curve b), AuNRs -HSA (curve c), AuNSs-HSA (curve d), (HSA) = 4.0 × 10^−6^ mol·L^−1^.

CD spectroscopy is one of the most commonly used methods to study the protein conformations in solution. Figure 7 shows the CD spectra of native HSA and three shape AuNPs-conjugated HSA solutions. The CD spectrum of HSA exhibits two negative minima in the ultraviolet region at 208 and 222 nm, which are the characteristics of an α-helical structure of the protein. It can be seen that when AuNFs were added, the characteristic peak intensities of the protein were reduced, but the shape and the position of the shoulder peak did not shift significantly. This phenomenon indicates that AuNFs only reduce the content of α-helical structure in protein secondary structure, but it still dominates. CD spectral measurements of HSA in the absence and presence of AgNPs were carried out in this work. The CD results were expressed in terms of mean residual ellipticity (MRE) according to the equations.

$$\text{MRE}=\frac{\text{Observed}\;\text{CD}\;\left(\text{mdeg}\right)}{\left[Cpnl\times 10\right]}$$ (1)

$$\alpha -\text{Helix}\left(\%\right)=\frac{-{\text{MRE}}_{208}-4000}{33000-4000}\times 100$$ (2)

Where C_p_ is the molar concentration of the protein, n is the number of amino acid residues (585 amino acids for HSA) and l is the path length of the cell (0.1 cm). MRE_208_ is the observed MRE value at 208 nm, 400 is the MRE value of *β*-form and random coil conformation cross at 208 nm, 33,000 is the MRE value of a pure α-helix at 208 nm. From the above equations, quantitative analysis results of α-helix content were obtained and shown in Figure 7. It can be seen that the α-helix content of native HSA showed an appreciable decrease from 52.1% to 47.7%, 35.1%, 27.2% upon its association with AuNFs, AuNRs and AuNSs, respectively. These results indicate that the binding of AgNPs with HSA caused a secondary structure change of the protein with a loss of helical stability. However, when AuNRs and AuNSs were added, the circular dichroism curve of the protein changed, and the shape and the position of the shoulder peak shifted significantly, indicating that the α-helical structure was not dominant. The above conclusion has also been confirmed for other nanoparticles. The research has confirmed that silver nanoparticles can also significantly change the conformation of HSA, reducing the α-helix content, which is consistent with our conclusion (Tian et al., 2022).

### 3.7 Fluorescence spectroscopy of the interactions between the AuNSs, AuNRs, AuNFs, and HSA

HSA alone exhibits strong fluorescence, but can also produce strong fluorescence when it interacts with AuNPs. In view of this, the interactions between the three types of AuNPs and HSA were measured using fluorescence spectroscopy, and the degree of fluorescence quenching of the solutions was used to determine the different degrees of binding. In this way, fluorescence spectroscopy was used to determine the different degrees of binding of the AuNPs with different morphologies and HSA to compare the properties of the three types of AuNPs (Park et al., 2019).

**FIGURE 8:**
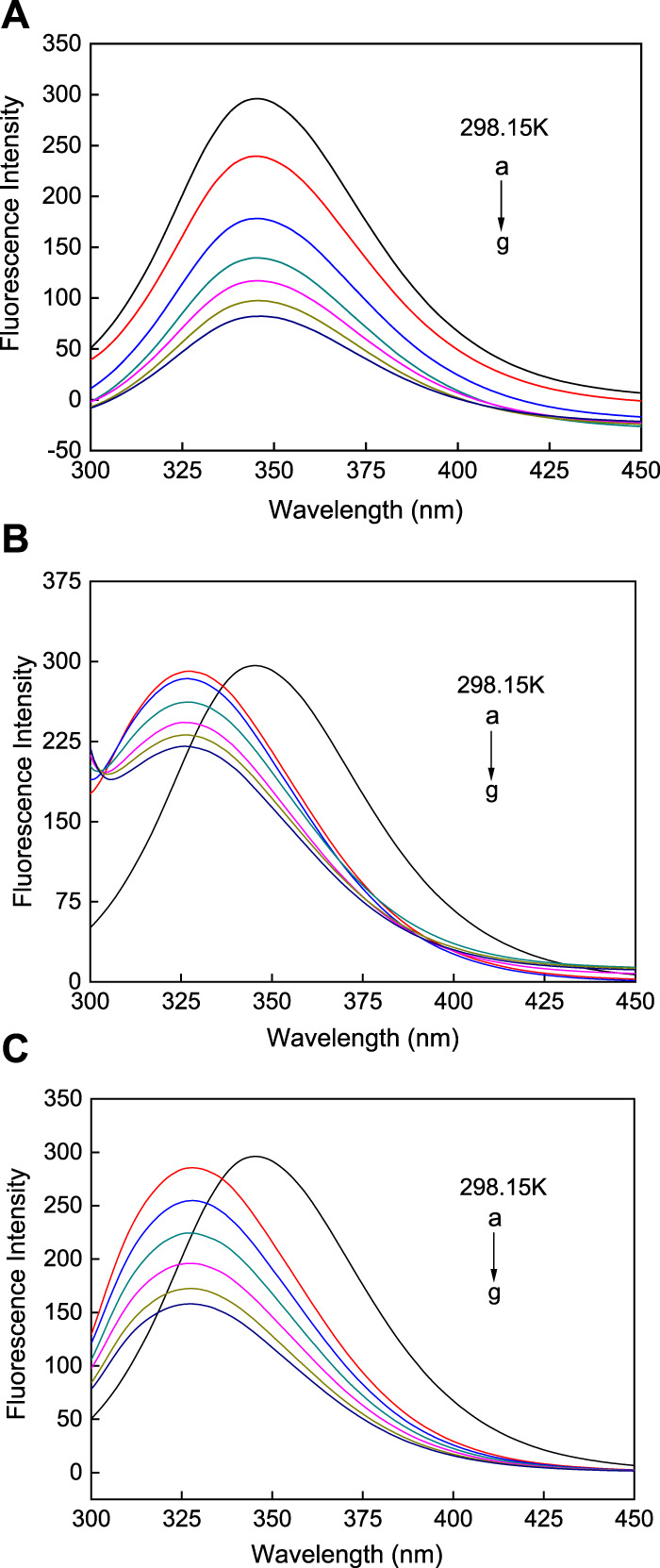
Fluorescence spectra of three different shapes of the AuNPs **(A)** AuNFs, **(B)** AuNRs and **(C)** AuNSs binding with HSA. The concentration of HSA was fixed at 3.00 μM; the concentration of the AuNPs from (a) to (g) is 0.00, 8.33, 16.70, 25.00, 33.30, 41.70, and 45.80 μM.

In Tris-HCl buffer at 298.15 K and a pH of 7.31, the AuNPs with different morphologies in a series of concentrations were reacted with HSA. As shown in Figure 8, when AuNP solutions of different concentrations were added to HSA, its fluorescence intensity decreased significantly, indicating that all three AuNP morphologies promoted the quenching of the fluorescence intensity of HSA, with a significant increase in the degree of fluorescence quenching of HSA with an increase in the concentration of the AuNPs. Under excitation at 280 nm, the fluorescence of HSA is mainly derived from tryptophan, therefore, the fluorescence quenching of HSA indicates a change in the tryptophan microenvironment. The AuNPs did not fluoresce, but when added to HSA stronger fluorescence quenching was observed upon an increase in the concentration of the AuNPs, indicating that all three types of AuNPs alter the structure of HSA. Therefore, this phenomenon may be caused by the interaction between the AuNPs and HSA. Fluorescence is mainly produced via aromatic exchange in the tryptophan group (electronic transition of a large conjugated *π* bond), and such an aromatic ring is hydrophobic. Based on this, it can be inferred that the association of the three types of AuNPs with protein molecules is mainly achieved via physical adsorption in the form of hydrophobic interactions. However, the quenching effects of the AuNPs with three different morphologies are different. When HSA binds with AuNFs, as the concentration of this AuNPs in solution increases, only promoted a continuous decrease in fluorescence intensity, with no occurrence of the blue shifting of the fluorescence peak (see Figure 8A). However, when HSA binds with AuNSs and AuNRs, the fluorescence intensity of HSA not only decreases, but also the fluorescence peak undergoes a blue shift (see Figures 8B, C). This phenomenon may be related to its quenching type, which will be analyzed in Section 3.10.

### 3.8 Analysis of synchronous fluorescence spectroscopy

**FIGURE 9:**
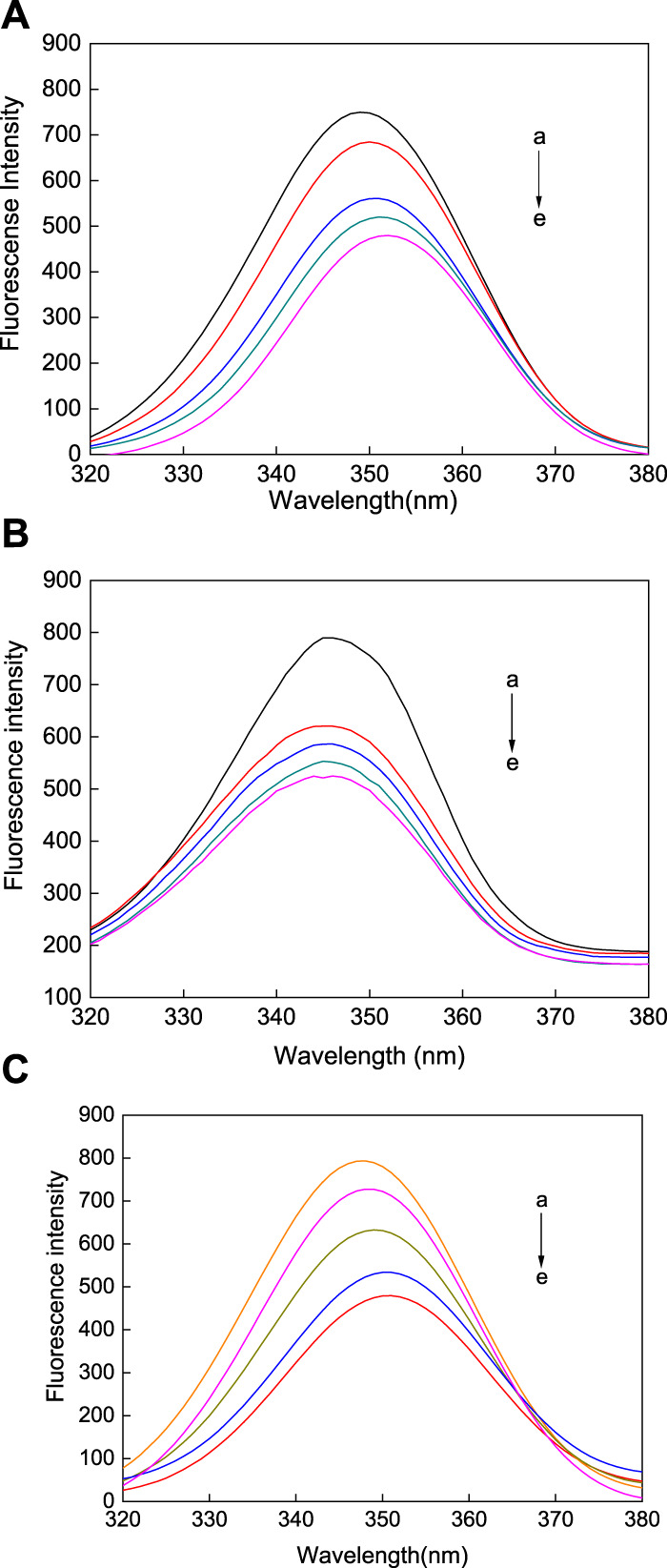
Fluorescence spectra of the **(A)** AuNFs, **(B)** AuNRs, **(C)** AuNSs binding with HSA when Δλ = 60 nm. The concentration of the AuNPs from (a) to (e) is 0.00, 8.33, 16.70, 25.00, and 33.30 μM.

**FIGURE 10:**
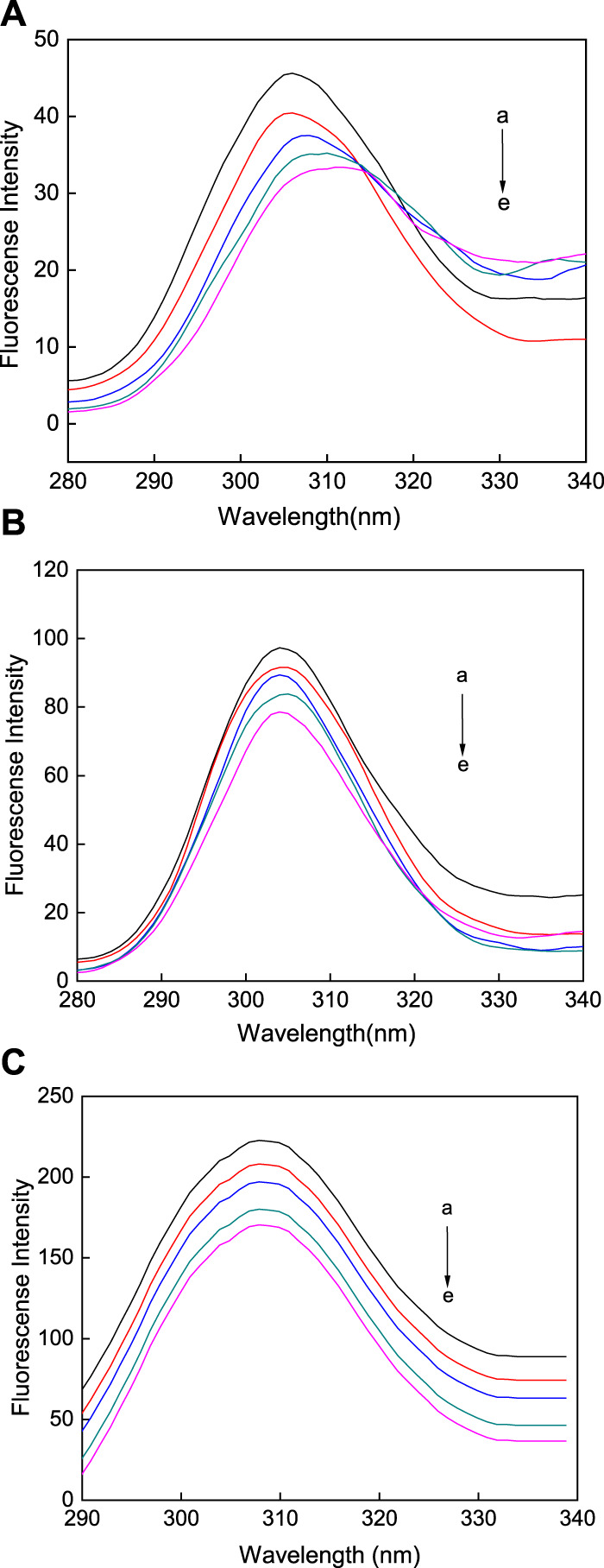
Fluorescence spectra of the **(A)** AuNFs, **(B)** AuNRs, **(C)** AuNSs binding with HSA when Δλ = 15 nm. The concentration of the AuNPs from (a) to (e) is 0.00, 0.35, 0.70, 1.40, and 2.80 μM.

Synchronous fluorescence spectroscopy is often used to study the conformational changes in HSA upon changes in external environment. Δλ represents the difference between the excitation and emission wavelengths. At a Δλ of 60 nm, the characteristic fluorescence of tryptophan is measured, however, when at a Δλ of 15 nm, the characteristic fluorescence of tyrosine is observed (Chakraborty et al., 2019). The synchronous fluorescence spectroscopy of tryptophan (Figure 9) and tyrosine (Figure 10) was measured in the reaction between the AuNFs, AuNRs, AuNSs, and HSA in a Tris-HCl buffer solution at a temperature of 298.15 K and pH of 7.31. It can be seen from Figures 9A−C that upon an increase in the concentration of the AuNFs, AuNRs, and AuNSs, the fluorescence intensity of the tryptophan residues decreases, and the fluorescence peaks of the AuNFs and AuNRs undergo red shifting. With an increase in the concentration of the AuNFs, the fluorescence intensity of the tyrosine residues decreases and the fluorescence peak is red shifted. Upon an increase in the concentration of AuNRs and AuNSs, there is also a decrease in the fluorescence intensity and no shifting of the fluorescence peak (Figures 10A–C). Therefore, it is concluded that HSA binds with the AuNFs, AuNRs, and AuNSs, resulting in changes in their fluorescence intensity.

### 3.9 Analysis of fluorescence quenching of HSA with AuNFs, AuNRs, and AuNSs

To account for the reduced emission intensity of HSA due to competitive absorption and reabsorption by AuNPs at the excitation and emission wavelengths of HSA, the inner-filter effect (IFE) should be considered. Therefore, the emission intensities were corrected for absorption of the exciting light and re-absorption of the emitted light to decrease the inner-filter effect before analyzing the data. The IFE corrections were applied using the equation from literature (Eq. 3):

FCorr=FObs×eAex+ Aem)/2 (3)

where F_Corr_ and F_Obs_ are the corrected and observed emission intensities, respectively. *A*ex and *A*em are the solution absorbance values at the excitation and emission wavelengths, respectively. The data obtained from corrected emission spectra were used for further analysis.

Fluorescence quenching denotes the process of the interaction between the quencher and the excited molecules of a fluorescent substance. To better understand the mechanism of the interaction between the AuNPs with the three different morphologies and HSA and compare the differences between them, variable-temperature fluorescence experiments were carried out at 303.15 and 308.15 K (Qin et al., 2010).

**FIGURE 11:**
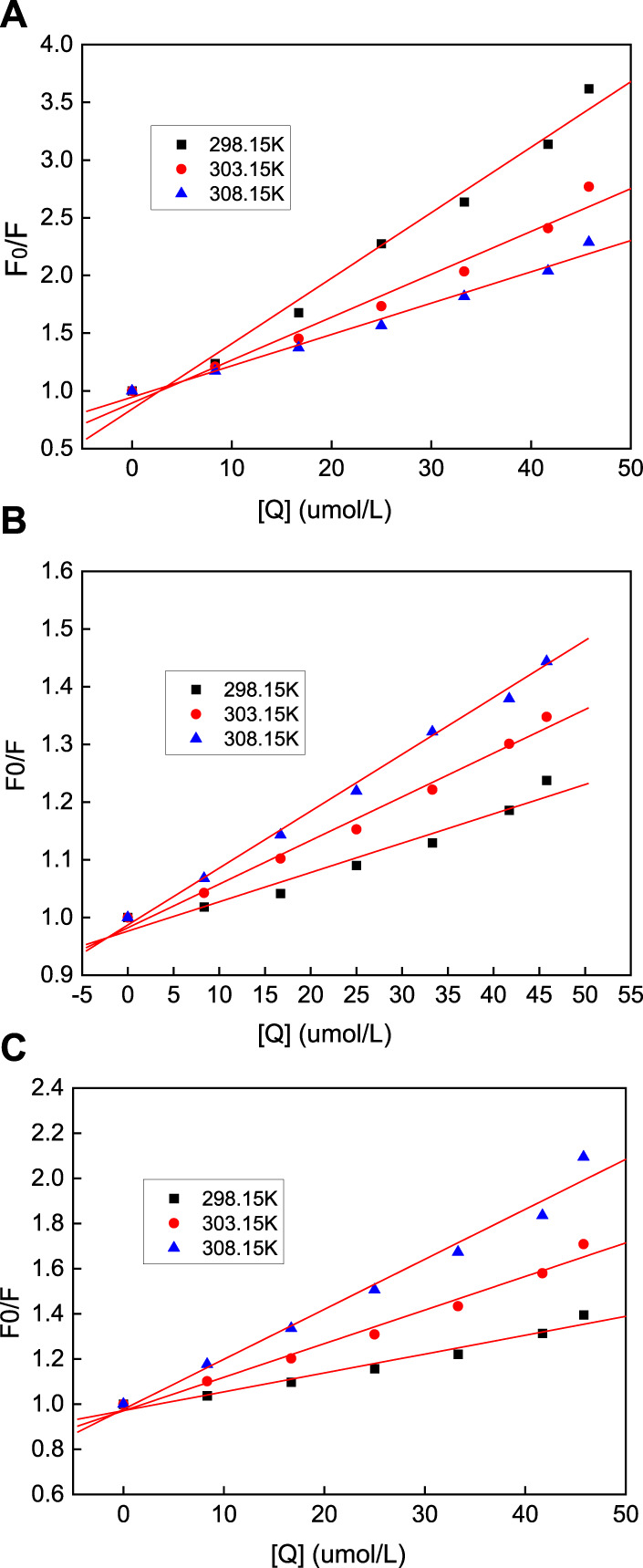
Stern-Volmer equation diagram of HSA quenching by nano-gold at different temperatures, where the **(A)** AuNFs, **(B)** AuNRs and **(C)** AuNSs.

Generally, the fluorescence quenching process can be divided into static and dynamic quenching, and the Stern–Volmer equation can be used to determine the quenching method:

$$F_{0}/F=1+K_{\text{sv}}\left[Q\right]=1+K_{q}\tau_{0}\left[Q\right]$$ (4)

where *F*_0_ is the fluorescence intensity of HSA when no AuNPs are added; *F* is the fluorescence intensity of HSA after the addition of AuNPs; *K*_sv_ is the Stern–Volmer quenching constant; *K*_q_ is the quenching rate constant; and τ_0_ is the average HSA life expectancy, with an average value of around 5.78 × 10^−9^ s. Using this equation, *F*_0_/*F* versus (Q) was plotted (Figure 11), from which the value of *K*_sv_ was determined. It can be seen from Table 1 that the *K*_sv_ value of the AuNFs decreases with increasing temperature, and that its *K*_q_ value is also much greater than the dynamic quenching constant under the maximum diffusion collision of HSA. Therefore, it can be concluded that the quenching effect that the AuNFs has on HSA is static quenching. It can be concluded from Table 1 that the *K*_sv_ values of the AuNSs and AuNRs increase in line with an increase in the temperature, but the *K*_q_ value is much greater than the dynamic quenching constant of 2.0 × 10^10^ (L·mol^−1^·s^−1^) under the maximum diffusion collision. Therefore, it can be judged that the quenching effect that the AuNSs and AuNRs has on HSA is mixed quenching.

**TABLE 1:** Stern-Volmer quenching constants of AuNPs-HSA system.

	*T*/K	*K*_sv_ (×10^3^L·mol^−1^)	*K*_q_ (×10^12^L·mol^−1^·s^−1^)
AuNFs + HSA	298.15	5.71 ± 0.08	9.86 ± 0.02
	303.15	3.71 ± 0.07	6.40 ± 0.01
	308.15	2.72 ± 0.02	4.67 ± 0.02
AuNRs + HSA	298.15	0.51 ± 0.03	0.88 ± 0.03
	303.15	0.76 ± 0.04	2.31 ± 0.05
	308.15	0.96 ± 0.05	1.67 ± 0.06
AuNSs + HSA	298.15	0.84 ± 0.02	1.44 ± 0.02
	303.15	1.49 ± 0.04	2.58 ± 0.01
	308.15	2.21 ± 0.05	3.81 ± 0.05

Time-resolved fluorescence measurements also have been introduced to investigate the quenching mechanism. The fluorescence lifetime of HSA in the absence and in the presence of AuNPs were measured at an emission maximum of about 340 nm (see Table 2). It can be seen that with the increasing concentration of AuNPs, there is no obvious change in the decay profile of HSA. An increasingamount of quencher did not significantly change the averagelifetime, suggestingthat the fluorescence quenching is mainly initiated by static quenching mechanism (Zhao et al., 2010). The research studied by Stefnao et al. also confirmed that the effect of gold nanoparticles on bovine serum albumin (BSA) is static quenching, which is consistent with our conclusion (Boulos et al., 2013). This result also supports the adsorption of HSA on thesurface of AuNPs and the formation of a ground state surfacecomplex.

**TABLE 2:** Fluorescence lifetimes data of AuNPs-HSA system at different concentration.

	C_AuNPs_(μM)	τ_1_(ns)	a_1_	τ_2_(ns)	a_2_	τ_AV_(ns)	χ^2^
Water + HSA	-	1,002	0.83	9,889	0.17	2,517	1.48
AuNFs + HSA	41.7	986	0.84	9,412	0.16	2,304	1.66
	83.4	1,052	0.86	9,983	0.14	2,319	1.82
	125.1	968	0.84	9,513	0.16	2,356	1.43
AuNRs + HSA	41.7	1,003	0.84	9,500	0.16	2,394	1.60
	83.4	995	0.84	9,618	0.16	2,345	1.61
	125.1	965	0.83	9,347	0.17	2,416	1.35
AuNSs + HSA	41.7	930	0.81	8,813	0.19	2,465	1.28
	83.4	969	0.82	9,291	0.18	2,464	1.24
	125.1	998	0.84	9,537	0.16	2,372	1.62

### 3.10 Thermodynamic data analysis

**FIGURE 12:**
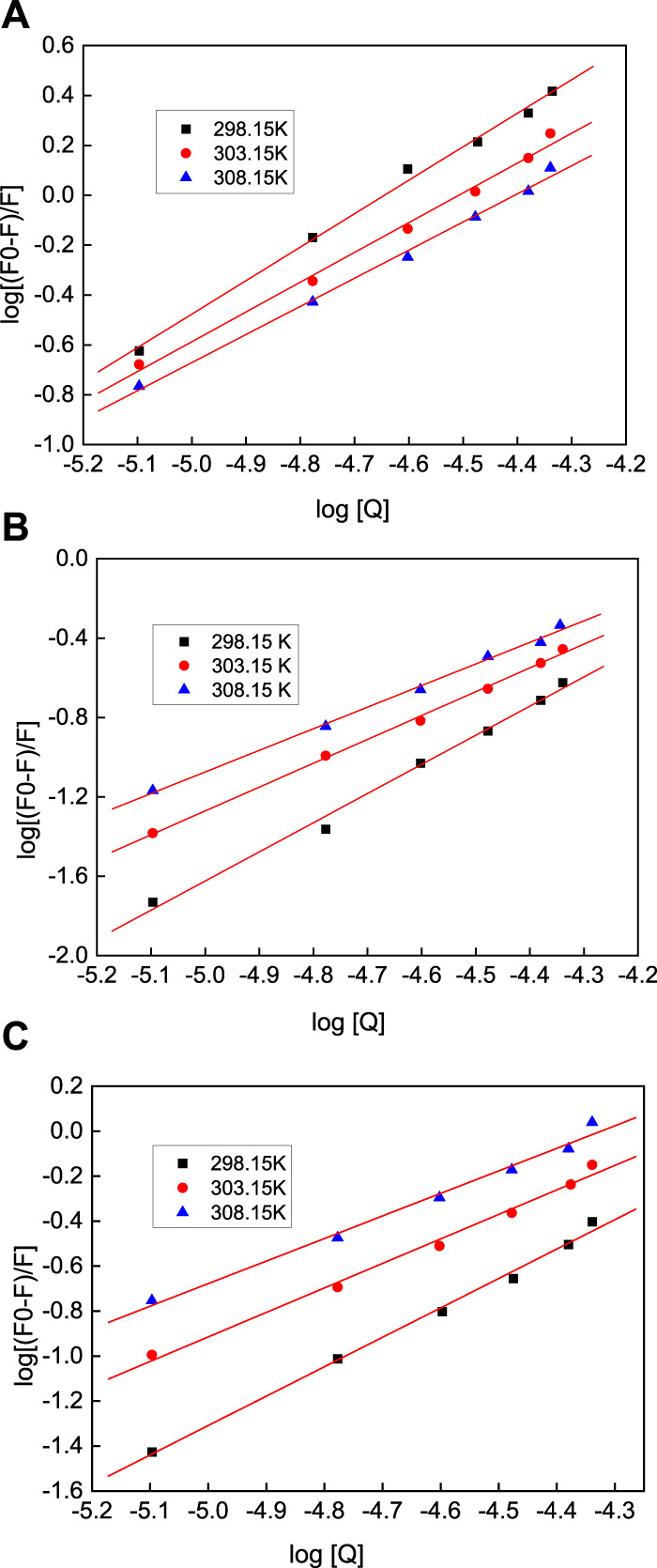
The double reciprocal equation diagram of the quenching of HSA by nano-gold at different temperatures, where the **(A)** AuNFs, **(B)** AuNRs and **(C)** AuNSs.

In terms of static quenching, to better understand and explain the interaction mechanism between the AuNPs with three different morphologies and HSA, the double-logarithm equation (Eq. 5) can be used to analyze the relationship between the fluorescence intensity and the quencher:

$$\log\frac{\left(F_{0}-F\right)}{F}=\log K_{a}+n\log\left[Q\right]$$ (5)

where *K*_a_ is the binding constant and *n* is the Hill constant. Figure 12 shows plots of log(*F*_0_ − *F*)/*F* vs. log(Q) (Ochnio et al., 2018; Hossain et al., 2020) of the quenching systems of the AuNFs, AuNRs, and AuNSs against HSA at different temperatures, which were plotted using to the double-logarithm equation The binding constant, *K*_a_, and Hill constant(*n*) of each system were also calculated, with the results presented in Table 3. By comparing the data of the AuNPs of the same morphology at different temperatures, it can be seen from Table 3 that although the AuNPs exhibit different morphologies, they all present a trend of decreasing *K*_a_ and *n* values as the temperature increases. That is to say, as the temperature increases, the AuNPs with three different morphologies exhibit lower binding of HSA. At the same temperature, the binding ability of the AuNPs of different morphologies toward HSA can be ranked in the order of: AuNFs > AuNRs > AuNSs. This result is consistent with the conclusions derived from the synchronous fluorescence measurements.

**TABLE 3:** Thermodynamic parameters of the AuNPs-HSA at different temperatures.

	*T*/K	*K*_*a*_ (×10^4^ L·mol^-1^)	*n*	Δ*H* (kJ⋅mol^-1^)	Δ*S* (J·mol^−1^·s^−1^)	Δ*G* (kJ·mol^−1^)
AuNFs + HSA	298.15	173.78 ± 0.03	1.34 ± 0.01	−223.79	−632.45	−35.21
	303.15	23.99 ± 0.11	1.19 ± 0.02			−32.05
	308.15	9.33 ± 0.02	1.13 ± 0.01			−28.89
AuNRs + HSA	298.15	52.48 ± 0.04	1.47 ± 0.03	−239.78		−32.09
	303.15	5.37 ± 0.02	1.20 ± 0.01		−696.60	−28.61
	308.15	2.29 ± 0.02	1.09 ± 0.02			−25.12
AuNSs + HSA	298.15	16.98 ± 0.03	1.31 ± 0.02	−158.76		−29.39
	303.15	3.39 ± 0.03	1.09 ± 0.01		−433.91	−27.22
	308.15	2.14 ± 0.04	1.00 ± 0.02			−25.05

Upon a small change in the temperature, the enthalpy change, Δ*H*, of the binding reaction can be regarded as a constant, and the corresponding thermodynamic parameters can be determined using the thermodynamic Eqs 6, 7.

$$\Delta G=\Delta H-T\Delta S=-\text{RTln}K_{a}$$ (6)

$$\ln\left(\frac{K_{2}}{K_{1}}\right)=\frac{\Delta H}{R}\left(\frac{1}{T_{1}}-\frac{1}{T_{2}}\right)$$ (7)

where Δ*G* is the change in the Gibbs free energy, Δ*H* is the change in enthalpy, Δ*S* is the change in entropy, and *K*_a_ is the binding equilibrium constant. According to the relative magnitude of the changes in the thermodynamic enthalpy and entropy before and after the reaction, the interaction types between the AuNPs with the three different morphologies and HSA can be determined. According to the relationship between the thermodynamic data and types of forces summarized by Ross et al., when Δ*H* > 0, Δ *S* > 0, there is a hydrophobic effect; when Δ*H* < 0, Δ*S* < 0, this indicates van der Waals forces and hydrogen bonding; and when Δ*H* < 0, Δ*S* > 0, this indicates electrostatic forces. The thermodynamic data of the interactions between the AuNPs with three different morphologies and HSA are shown in Table 3, from which it can be seen that the three types of AuNPs exhibit Δ*G* < 0. That is, their interactions with HSA are spontaneous reactions. For Δ*H* < 0, Δ*S* < 0, it can be inferred that the types of forces between the three types of AuNPs and HSA are mainly van der Waals forces and hydrogen bonds.

### 3.11 3D fluorescence spectroscopy

**FIGURE 13:**
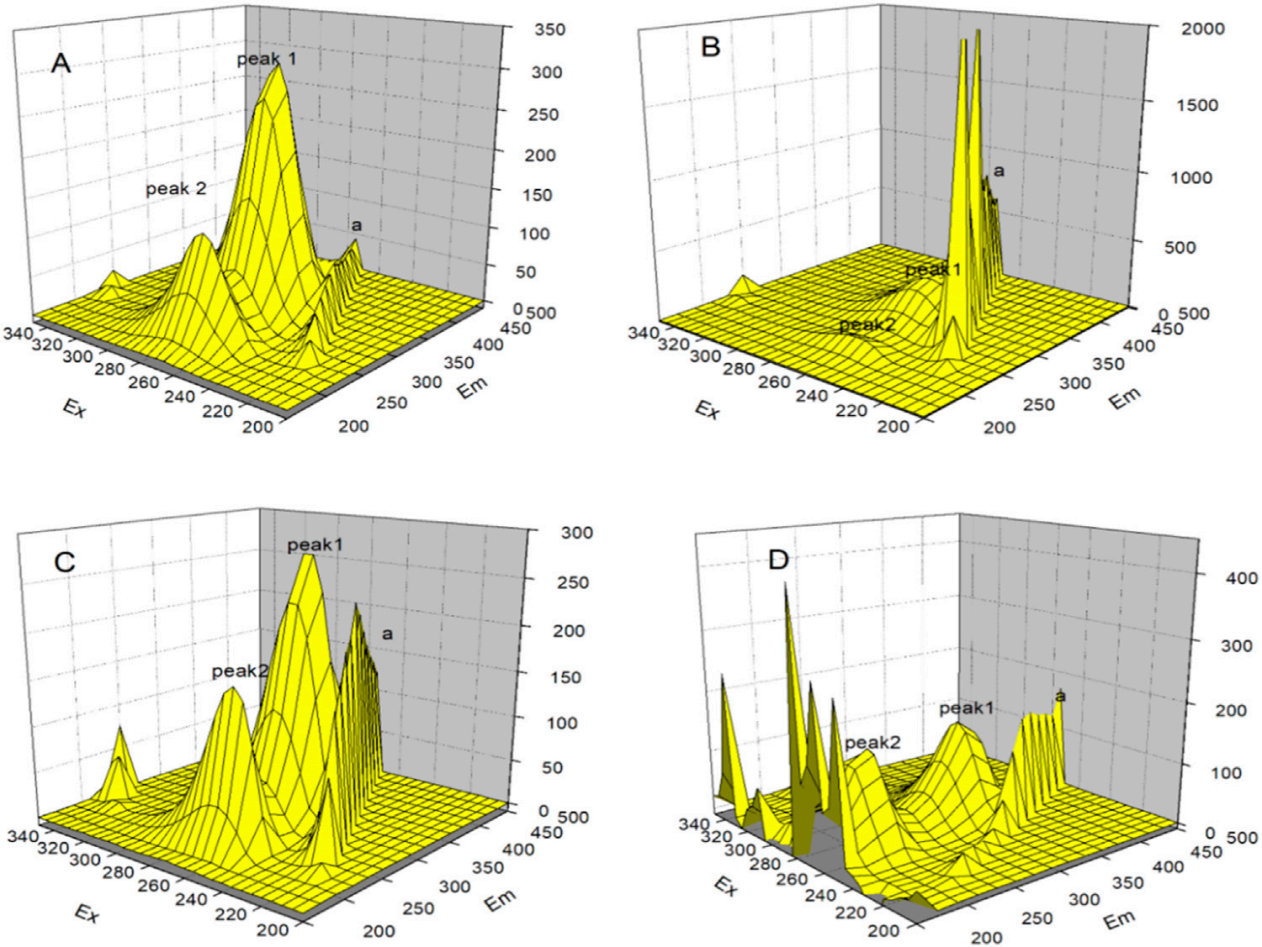
3D fluorescence spectra of the interaction between gold nanoparticles and HSA, in which **(A)** blank HSA, **(B)** AuNRs, **(C)** AuNSs and **(D)** AuNFs.

The three dimensions of 3D fluorescence spectroscopy are the excitation wavelength, emission wavelength, and the corresponding fluorescence intensity. The peak position and peak intensity of the fluorescence spectra, as well as the changes in the characteristics of the absorptions in the spectra can be visually observed from Figure 13. 3D fluorescence spectroscopy experiments allow further understanding to be gained of the interactions between the three types of AuNPs and HSA. Figure 13A shows the fluorescence spectrum of pure HSA, wherein peak position 1 at λ_ex_: 280 nm, λ_em_: 340 nm is the characteristic peak of HSA, and mainly due to the spectral characteristic maps of the tryptophan and tyrosine residues and peak position 2 at λ_ex_: 230 nm, λ_em_: 340 nm is related to the polypeptide backbone of the protein; and the peak position at a(λ_ex_ = λ_em_) is the Rayleigh scattering peak. Figures 13B−D show the fluorescence spectra of the mixed solutions with certain concentrations of the AuNRs, AuNSs, and AuNFs. Upon the addition of the three types of AuNPs, the fluorescence intensities of peaks 1 and 2 are reduced, while the intensity of peak a is increased. The results of this experiment further show that the three types of AuNPs interact with HSA and have the effect of changing the structure of its protein peptide chain.

### 3.12 Molecular docking studies

**FIGURE 14:**
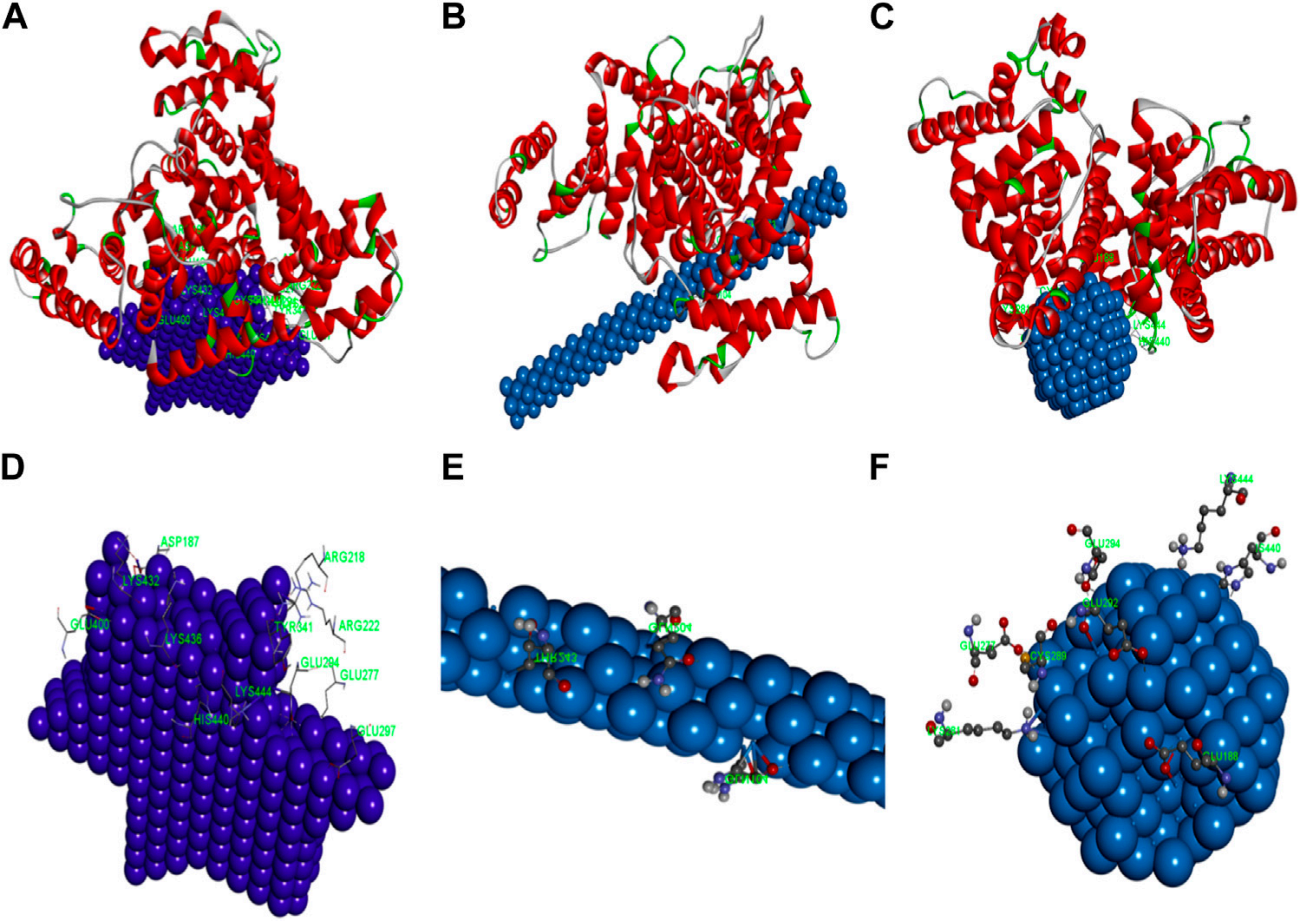
The simulation diagram of the molecular docking between HSA and **(A)** AgNFs, **(B)** AgNRs, **(C)** AgNSs. The spiral is HSA. The detailed map of **(D)** AgNFs, **(E)** AgNRs, **(F)** AgNSs and surrounding amino acid residues.

Molecular docking studies were finally performed to identify the nature of the binding process between AuNPs and HSA molecules. The stable configurations of HSA adsorbed on the surfaces of AuNPs are displayed in Figures 14A−C. The docked complex structures of AuNPs and the surrounding amino acid residues within the radius of about 3 Å were analyzed. The amino acid residues with a distance of less than 3 Å between HSA and AuNFs are ASP187, ARG218, APG222, GLU277, GLU294, GLU297, TYR341, GLU400, LYS432, LYS436, LYS444, HIS440 (see Figure 14D); that of AuNRs are GLN104, GLN204, THR243 (see Figure 14E); and that of AuNSs are GLU188, LYS444, GLU292, GLU294, LYS281, HIS440 (see Figure 14F).

## 4 Conclusion

In this study, AuNPs with three different morphologies (AuNFs, AuNRs, and AuNSs) were synthesized via a chemical method and characterized by UV-vis absorption spectroscopy, and TEM. The TEM results confirmed that the three AuNPs synthesized by the above synthesis method have their own specific morphology, namely,: flower shape, rod shape, and spherical shape, and the particle sizes are 42.3 ± 1.4, 40.3 ± 1.8, and 20.0 ± 0.3 nm, respectively. At the same time, they also have good dispersion. The above results have also been verified in DLS and Zeta Potential Measurements. Using UV-visible absorption, DLS, Zeta potential measurements, fluorescence spectroscopy, FTIR, CD spectroscopy, molecular docking studies, and 3D fluorescence spectroscopy to compare the results before and after the interaction between three different shapes of AuNPs and HSA, all can prove that they interact with HSA. It is worth noting that the FTIR measurement results show that all three AuNPs can induce changes in the secondary structure of HSA. The order of this ability from small to large is AuNFs, AuNRs, and AuNSs. It was verified again by CD spectroscopy, and the results were the same as above. The fluorescence spectroscopy results showed that all three types of AuNPs undergo a quenching reaction with HSA, and that the quenching mechanism of the three materials is static quenching. All three types of AuNPs change the polarity of the hydrophilic environment around the tryptophan residues in HSA, but the peak positions of the AuNSs and AuNRs are blue shifted, while the peak position of the AuNFs does not change. From the analysis of the 3D fluorescence spectra, it can be seen that all three types of AuNPs interact with HSA and change its peptide chain structure. It can also be seen from variable-temperature fluorescence spectroscopy measurements that the binding constants, *K*_a_, of the three types of AuNPs decrease with an increase in temperature, indicating that their binding ability decreases with an increase in temperature. However, at the same temperature, the AuNFs exhibit higher *K*_a_ values than the AuNRs and AuNSs, indicating that the AuNFs exhibit stronger binding ability toward HSA than the AuNRs and AuNSs.

The performance of metal nanomaterials is significantly influenced by the size, composition, crystallinity, shape, and structure of the particles. The properties of nanoparticles might theoretically be accurately controlled by varying the aforementioned qualities. Morphology control is the aim of research on nanomaterial preparation. Consequently, the production of metal nanoparticles with customizable forms and unique morphologies is one of the current research hotspots for scientists. In our investigation, we contrasted the interactions of AuNFs, AuNRs, and AuNSs with HSA. AuNFs interact more favorably with HSA, according to studies. This can be used as a reference for the administration of drugs containing AuNPs.

## Data Availability

The raw data supporting the conclusion of this article will be made available by the authors, without undue reservation.
